# The State of Lunacy in Ireland
*The Ninth Report of the District, Criminal, and Private Lunatic Asylums in Ireland. Blue Book. 1859.


**Published:** 1860-01-01

**Authors:** 


### Art. VII.—THE STATE OF LUNACY IN IRELAND.*

Twelve months ago, when examining the Report of the Royal Commission, appointed in 1856 to inquire into the state of Lunacy affairs in Ireland, we had occasion to express the opinion that the Commissioners had dealt but scanty justice towards the asylums of that portion of the kingdom, and their management. It appeared to us that the Commissioners had adopted a standard of comparison which was scarcely applicable under the circumstances, and that, in consequence, existing and presumed faults were presented in a somewhat exaggerated light. We turned, therefore, with more than ordinary interest, to the first Report which has been published by the Irish Lunacy Inspectors, since the appearance of the Report of the Royal Commissioners; for we expected to learn much from the former Report in confirmation or correction of the opinions we had formed concerning the latter one. We have not been disappointed, and we are glad to add that our favourable impressions are abundantly supported.

The present Report of the Irish Lunacy Inspectors is not merely of local interest; it is a most carefully digested work, of much practical and scientific value, apart from its more immediate object. It admirably fulfils its special purpose, and yet it claims the attention of all persons interested in the welfare of lunatics, and who are wishful to advance our knowledge of mental alienation. The statistical Appendix to the Report is of great interest, and the tables may be instanced as types of asylum returns.

The Report refers to the two years ending 31st March, 1859, and from it we learn that the working of the asylums during that period has been highly successful, as evinced by a greater number of recoveries in them, and by a less mortality than has been observed elsewhere in kindred institutions.

In Ireland, as in other parts of the kingdom, the great difficulty to be contended with is the provision of increased accommodation for the care of the lunatic poor. The asylums are choked with chronic cases, and the space at disposal for recent ones is necessarily brought within a narrow verge, while there is still outside the asylum walls a large lunatic population for whom accommodation is required. The asylums opened within recent years have not been sufficient in number to effect much change in this state of affairs, although sensibly relieving the localities in which they have been erected, from the more pressing necessities felt with regard to their lunatic inhabitants. There is, in fact, a great deficiency of asylum accommodation in Ireland, and this throws serious, and, in some instances, insuperable difficulties in the way of effective improvement in the state of the floating lunatic population. The defects experienced in the provision for the insane are, indeed, similar in kind, but differing in degree, from those felt in England and Scotland, and the great problem with the Irish Lunacy Board, as with

* The Ninth Report of the District, Criminal, and Private Lunatic Asylums in Ireland. Blue Book. 1859.

the English and Scotch Boards, is the most practicable mode of providing increased asylum accommodation. In Ireland, however, the stress is more severely felt than in other portions of the kingdom, and it is requisite to have recourse to the gaols for the security of many lunatics. In some instances, as in both ridings of Tipperary, the prisons contain almost as many lunatics as ordinary convicts.

Asylum management, and the care of lunatics generally, under circumstances such as these, are questions of considerable complexity, and it redounds in no small degree to the credit of the Irish Inspectors, that they have been able to effect, year by year, a steady and peristent improvement in the condition of the insane.

The chief aim of the Inspectors, for the present, seems to be centred in the provision of new asylum accommodation; for without this, any further marked improvement in the condition of the lunatic poor cannot well be anticipated. Regarding the nature of the accommodation required, their views coincide with those of the English and Scotch Commissioners. They would divide the insane into two classes. In the first, they would include urgent and curable cases, as well as cases which, though not admitting any reasonable hope of recovery, still require peculiar treatment, whether from dangerous tendencies, violence, or peculiarity of habits; in the second, they would include the idiotic, the great majority of epileptics, and the domestic, whose mental and corporeal powers decline *pari passu*, but who cannot be rightly cared for except in establishments solely devoted to their use. For the former a more expensive organization is required in regard to staff, building, and appliances; for the latter plan, airy, inexpensive, but commodious buildings, with ample means of occupation, both in and out of doors, would be sufficient.

In what manner these suggestions might be best carried out is carefully and clearly considered by the Commissioners, the obstacles lying in the way being fully noted. This the most important portion of the Report in its local bearing is of least interest to the English reader, as it would require a certain degree of familiarity with local, fiscal, and territorial arrangements, in order rightly to appreciate the details given by the Inspectors. There seems to be good reason for hoping that the principles of improvement laid down, and the legislative changes which would be requisite for their fulfilment, will be eventually carried out.

One very important piece of knowledge possessed by the Inspectors is this: an accurate acquaintance with the sum total of lunacy, and of the condition of all the lunatics in Ireland,* so that the amount of additional asylum accommodation required may be calculated with precision.

It is worthy of remark, that on account of the unbroken prosperity of Ireland for some years, together with a diminished agricultural population, many union workhouses have become comparatively untenanted. It has been proposed to make these buildings available as asylums for the idiotic and epileptic. Again, it has been suggested that plain, substantial buildings should be erected for the class of

* See Vol. XI. of this Journal, p. 107.

patients named, in close proximity to existing asylums. By this arrangement one staff could take the charge of both buildings, at a considerable saving of expense, and many improvements be effected in common. The Commissioners think that the first proposition could be carried out with advantage in a few instances, it being understood that the house should be entirely appropriated to lunatics, and properly fitted up for them. They doubt whether this scheme would be as economical as it appears, as the buildings would have to be gutted and refitted properly, and the expense would doubtless be little inferior to the better scheme of erecting special buildings contiguous to other asylums.

On the whole, it would appear that the current rate of asylum expenditure in Ireland is less "(probably by 30 per cent.) than what obtains generally throughout England, where, no doubt, the interior fittings and arrangements of hospitals for the insane being adapted to the habitual comforts of its population, are more expensive, but, considering the social condition of the two countries, not affording to their inmates greater relative advantages. It cannot, therefore, but be gratifying to us [continue the Inspectors] to be enabled to assure your Excellency that while all our public asylums are steadily progressing, and from day to day assuming, from in and out door improvements, an air of neatness and of culture, many of them, in point of cleanliness, regularity, and order, notwithstanding the domestic deprivations and former mode of living of three-fourths of the patients, are highly commendable. In some institutions a more liberal spirit pervades the minds of the governors, particularly in regard to articles which cannot perhaps be said to be absolutely necessary to the well-being of the inmates, but the granting of which materially tends to both their comforts and amusements."—(P. 8.)

The total number of patients under treatment in the district asylums, during the two years, amounted to 10,420. Of these, 594 died, and 1738 were discharged, of whom 1267 had recovered, and 345 were improved. The proportion of cures during the same period, calculated upon admissions, amounted to 48·71 per cent., as against 36·99 in Scotland, and 38·49 in England. Independent of the recoveries in the asylums, "there were also discharged, improved, 13·26 per cent. of the admissions, representing, under both heads, 62 per cent. Of the daily average, 16 per cent. recovered, and 4·20 improved; and in like manner, on the total under treatment, 12·15 and 3·30 respectively."—(P. 11.)

The figures also tell in favour of the Irish asylums, if they be calculated upon the daily average number under treatment, or on the total number in hospital during the year, as well as on the admissions. It would appear also that the average of cures in the Irish asylums is "nearly three per cent., or, in the abstract, relatively speaking, almost a fifth over those of France."—(P. 11.)

The mortality in the district asylums during the last two years amounted to 7·42 per cent., as compared with 8·37 in the Scotch, and 10·30 per cent. in English asylums. In this respect the Irish district asylums have again the advantage. Eight of the deaths were of a suicidal, and one of a homicidal, nature. "Heretofore such fatal occurrences as those just stated, or casualties terminating in the loss of life, have been almost unknown in district asylums, averaging for the last eight years little more than two annually, or one in the proportion of every 2300 patients under treatment."—(p. 11.) The annual average of deaths from violence and suicide in English asylums during four years, as reported in 1857 and 1858, amounted to 31. The proportion to patients under treatment is not given, but the Commissioners remark:—

"It may appear extraordinary that in an excitable race, as the Irish are generally reputed to be, deaths by violence should be so few. It may probably be owing to the circumstance that the more openly dangerous and uncontrollable the patients, the more they are watched. We have reason to believe that the parties who attempt to injure themselves or others, being apparently the most tranquil and amenable, are frequently allowed a greater latitude of freedom than their companions in confinement—hence too much care cannot be employed in the selection of persons of intelligence as attendants on the insane, who, to guard against accidents, require unceasing supervision."—(p. 12.)

The Commissioners also add, in reference to the comparative statistics they have given, the following observations:—

"It is, we trust, unnecessary to state that, in making the preceding observations we are not influenced by a desire to draw any invidious contrasts, being fully aware how much the success of asylum management depends on circumstances, and that no professional or moral treatment, however judicious, can overcome certain forms of mental disease. Still it may be pardonable in us to compare generally the results obtained in the asylums of other countries with those under our immediate control, and refer thereby to the more successful issue of the latter, and their consequent utility; not that we mean to deny the existence in them of numerous faults and imperfections, but which it is to be hoped will gradually disappear." (p. 12.)

In reference to the assigned causes of insanity, the Commissioners state that of 2003 cases, no less than 755 were traceable to hereditary transmission or intemperance. They also remark that moral predominate over physical causes among women, adding in reference to puerperal mania, that they are disposed to regard this form of insanity as being "almost as much a matter of legal as of medical investigation, from the fact that no inconsiderable proportion of the cases registered on the books of asylums under that denomination had been already subjected to inquiry in courts of justice." (p. 12.)

Illustrative of the degrees of relationship existing at times between lunatics in asylums, the following facts are mentioned. Within the two years there were admitted into the Limerick Asylum ten, and into the Waterford six individuals having the relationship of brothers and sisters; and into the Carlow Asylum nineteen persons in the relation of first-cousins.

The sanitary condition of the Asylums was very favourable throughout the two years. The cases of relapse amounted to about one in six of the admissions. "They were not, however, from recoveries within any particular period, many of them having been discharged from asylums from three to ten years before. We mention this circumstance as showing the liability to a relapse after a long interval of sanity. The longest that has come officially within our knowledge was eighteen years, during which the party enjoyed the best health both mental and bodily. The second was a severe and protracted attack, followed, however, by a perfect recovery." (p. 13.)

The Commissioners lay stress upon the advantages derived from religious ministrations in the asylums, and point out the necessity which exists for legislation in order to secure the same.

The bill providing for the superannuation of managers and servants works well, and the non-professional superintendents of asylums have been gradually superseded by professional men, one of the former only now remaining. The Commissioners urge the importance of establishing clinical instruction in the asylums; and look upon the appointment of visiting Physicians as important, particularly from giving confidence to the public in the management of these institutions. The success arising from the encouragement of literary pursuits in the Irish Asylums has not been great, from the deficiency of preliminary instruction among the patients; prints, pictures, and ornaments attached to the walls of the corridors are, however, found to be to the taste of the inmates.

The poor-houses, as with us, are stated to be unsatisfactory domiciles for the insane, and for the same reasons; and many disadvantages arise from the committal of lunatics to gaols. The Central Criminal Asylum works well; and speaking of the general habits of the patients, the Commissioners say:—

"It may be remarked that they scarcely ever allude to their own offences, or to the nature of the institution itself, by drawing a distinction between it and others. Strong objections exist, and perhaps justly, to convicted thieves and felons, who become insane in prison, being mixed up with ordinary lunatics in County Asylums, as the *morale* of the institution, and the self-respect of untainted parties, might be injuriously affected by the association. Even were there no other reason, from this consideration alone we would recognise the advantages of a central establishment such as that at Dundrum. No doubt individuals committing offences very different in degree are detained in it, but practically no inconvenience has arisen therefrom. The lesser and greater criminals, if criminals they be, meet together, and never feel themselves so exempt from blame as to cast reproach on others. The sane no doubt suffer from a protracted intercourse with their maniacal and idiotic companions, an inconvenience which can only be obviated by fostering the hope that though their detention is rendered necessary by a due regard to public prejudice and the public safety, their liberation is to be dependent upon their own good conduct. The principal difficulty to contend with arises from a perversity of disposition displayed by convicts, principally on admission. They give a bad example to their fellows, so much so, that the resident physician would, if the arrangement of the house admitted it, have them placed apart in probationary wards—a practical suggestion which may be of much value in the future construction of similar establishments."—(p. 21.)

The condition of private asylums is reported to be in several instances very creditable, in others much improved. The mortality in these establishments during the two years has been remarkably low, amounting only to 6·50 per cent. on the average under treatment; and the proportion of recoveries very satisfactory, being 36·59 per cent.

as calculated upon admissions. In two instances only have special investigations been necessary, and in both they had reference to the escape of patients. The sources of complaint by the Commissioners respecting patients in private asylums rest principally on the same grounds as with us, to wit, the neglect exhibited towards patients by relatives; want of sufficient care in filling up certificates by medical men, even in well-marked cases; and the inability of permitting patients to leave the asylum on probation. The want of accommodation for persons of limited means is also much felt. The Commissioners direct attention, moreover, to the anomalous state of the law regarding lunacy inquisitions, and suggest a modification of the same.

In concluding our notice of this Report we would again direct attention to the valuable statistical appendix. We shall not enter into any details upon this portion of the Report, as comparatively recently we had occasion to present to our readers a summary of Irish Lunacy Statistics, and we shall, sooner or later, have to recur to the subject. We would, however, point out a defect in the tables which we think might be readily remedied in the preparation of other Reports, adding thus to their value, as well as to the comfort of readers. We allude to the want of summaries of the different tables, as well as of a summary of the results of previous years. By the latter addition a comparison of results would be practicable, accuracy would be secured, and the scientific value of the tables increased; by the former much labour would be saved which is now needlessly entailed upon readers, and which might be avoided by a slight additional expenditure of type on the part of the printer, and of calculation or writing on the part of the compiler.

From the whole of the Report we conclude that there is a steady and most gratifying improvement in all that relates to lunacy matters in Ireland, with one exception. The prime workers in the improvement, and the men upon whom it most depends, seem to have been forgotten; we allude to the Inspectors in Lunacy. Their salaries are quite incommensurate with the duties they perform, and as compared with those attached to several legal posts of secondary importance in the Irish Government, or with those of our own Commissioners in Lunacy, they are most parsimonious. We have had to allude to this subject on previous occasions, and we regret to find that there is still no appearance of justice being done in this respect to the Irish Inspectors in Lunacy.

